# An Unusual, and Probably Unique, Complication (General Emphysema) of Tracheotomy

**Published:** 1901-08

**Authors:** E. C. Ellett

**Affiliations:** Memphis, Tenn.; Ophthalmic and Aural Surgeon to St. Joseph’s Hospital, the Children’s Home, the Lucy Brinkley Hospital, and the City Hospital


### AN UNUSUAL, AND PROBABLY UNIQUE, COMPLICATION (GENERAL EMPHYSEMA) OF TRACHEOTOMY.

By E. C. ELLETT, M.D., Memphis, Tenn.

Ophthalmic and Aural Surgeon to St. Joseph's Hospital, the Children's Home, the Lucy Brinkley Hospital, and the City Hospital.

The following case was observed about three years ago, and at that time and on several subsequent occasions I have attempted to find some record of a similar result following the operation of tracheotomy. Besides a careful search of what literature I had in my own possession or could gain access to, I went carefully through the catalogue of the library of the Surgeon-General's office and had sent to me some fifteen or twenty volumes containing matter which might bear on the subject. In addition to this I have mentioned the case to others of some experience in this work, but I have been unable to learn of any other case in which the condition appeared to anything like the extent it did in mine.

The following are the notes of the case:

The patient was a boy two years old, whom I saw through the kindness of Dr. Rudisill on July 27, 1898. Four days before, while eating a watermelon, he was seized with an attack of coughing, quite croupy in character, which had continued ever since, the child presenting no other symptoms. Auscultation over the trachea elicited a sound which might have been produced by the flapping of a light foreign body. Expectant treatment not being agreeable to the family, tracheotomy (the low operation) was done next day under chloroform. Aside from the embarrassment occasioned by a short fat neck, the operation was an ordinary tracheotomy.

Exploration of the trachea down to the bifurcation and up to the larynx did not detect any foreign substance, but induced a fit of coughing by which a watermelon seed was expelled from the wound. The wound was then cleansed and closed with three layers of catgut sutures, one in the trachea, one in the muscles and one in the skin and fascia, and the dressings applied. Following recovery from the anesthetic, the child began to cry, forcing air out of the tracheal wound, and thence along the layers of cervical fascia, since close apposition of the skin wound prevented its escape to the surface. The child swelled to an alarming degree, the emphysema being at first confined to the left side of the body and the right side of the head, but soon involved the subcutaneous tissue of neck, face, scalp, chest, abdomen and scrotum. The legs were not affected and only one arm (left) slightly. In a crying spell the child would double his size, and on quieting the swelling would greatly subside, but not disappear. In some places the skin was raised nearly two inches from the underlying parts. The next day the condition was less marked, and after six days was about gone. The wound healed by first intention, and the child was at play three days after the operation. The appearance was one which, to a person unacquainted with the cause, was truly alarming. At its appearance the parents of the child sent in haste for Dr. Rudisill, who recognized the condition at once and soon quieted their fears.

The case was reported to the Memphis Medical Society in August, 1898, and in the discussion Dr. Sale mentioned a case similar in many respects upon which he had operated some years before. Emphysema of the chest and neck developed without any ill effect.

Randolph Building.

